# Understanding the Needs of Veterans with PTSD

**DOI:** 10.3390/healthcare6030100

**Published:** 2018-08-15

**Authors:** Dominic M. Murphy, Walter Busuttil, David Turgoose

**Affiliations:** 1 Research Department, Combat Stress, Leatherhead, Surrey KT22 0BX, UK; Walter.Busuttil@combatstress.org.uk (W.B.); David.Turgoose@combatstress.org.uk (D.T.); 2 Department of Psychological Medicine, King’s Centre for Military Health Research, King’s College London, London WC2R 2LS, UK

**Keywords:** veterans, military, ex-service personnel, PTSD, trauma, treatment

Each year significant numbers of individuals leave military service. The majority successfully transition into civilian life, but a significant minority leave the military either with a diagnosis of PTSD, or go on to report symptoms in later life. Prevalence rates of PTSD in military populations have been extensively studied since the deployments to Iraq and Afghanistan, with data suggesting high rates (in some cases up to 20%) in those who served in these conflicts [1]. There is also evidence that, with the passage of time since individuals return from deployment, rates of PTSD increase [1,2]. PTSD has been shown to create a higher financial burden to society than other mental health conditions in veterans [3].

At the same time, there is a body of research showing that only a minority of veterans with PTSD are able to engage in help-seeking behaviours [4]. In addition, it has been noted that members of the military are less likely to attend health appointments for mental health needs compared to physical health needs [5]. Practical access barriers (such as lack of awareness of services), and those barriers related to stigma, have been associated with lower levels of help-seeking [6]. Evidence suggests that veterans with mental health difficulties are more likely to report a greater number of barriers to seeking help than those without mental health difficulties [6,7,8]. This relationship between mental health status and endorsing more barriers to engaging in support is also evident in the partners of veterans [9].

A range of different factors have been identified that could contribute to mental health difficulties in veterans. These include pre-service factors such as growing up in areas of social deprivation or reporting difficult childhood experiences [10,11]. Factors related to military service such as having served in the army, being involved with direct combat exposure, being from the lower ranks, alcohol misuse and leaving the military prematurely (often referred to as being an early service leaver) have all been associated with an increased risk of reporting mental health difficulties [12]. After leaving the military, a lack of social support, taking longer to seeking help, unemployment and relationship difficulties have also been reported as risk factors for experiencing mental health difficulties [13].

A further consideration is the evidence suggesting that veterans do not respond as well to treatment for PTSD as non-veteran groups do [14,15,16]. The ICD-11 has recently been published and for the first time includes a diagnosis of complex PTSD (cPTSD). It could be advantageous to explore the relative frequency of cPTSD and PTSD prevalence rates in veteran populations and the impact of meeting criteria for cPTSD and response to current treatments. This may provide evidence for why veterans with PTSD appear to respond less favourably to interventions than other populations and highlights the need for the development of new methods to support this vulnerable population.

This special edition of *Healthcare* features original papers exploring a range of divergent issues surrounding PTSD in veterans and discusses novel ways to support this vulnerable population. For example, Poulsen et al. published a paper exploring the application of nature-based therapy (NBT) for veterans with PTSD [17]. They presented qualitative data that reviewed the benefits of a novel 10-week programme that employed NBT delivered within a therapy garden. Promising early evidence was found to support the use of NBT, in particular, for improvement in functional impairment.

Ashwick et al. published a paper looking at the relationship between mental health and risk-taking behaviours within a sample of veterans engaged in clinical mental health services [18]. They reported high rates of risk-taking (e.g., driving above the speed limit, illicit drug use, etc.) within this population. PTSD was observed to be independently associated with risk-taking behaviours. In particular, the DSM-5 PTSD symptom clusters of hyperarousal and elevated negative alterations in mood and cognition predicted risk-taking behaviours. This provides evidence for the importance of screening for risk-taking behaviours in veterans with PTSD.

The same research team as Turgoose et al. also published a paper within the special edition that examined the associations between anger, aggression and PTSD in veterans [19]. Whilst the high prevalence of anger in veterans is well documented in the literature, less is known about its relationship to PTSD in clinical samples. Both anger and aggression were observed to be more prevalent in individuals who report high rates of childhood adversity and were associated with a higher burden of PTSD symptoms.

Another paper within this special edition looked at potential bias by jurors faced with possible malingering for PTSD in veterans involved with the judicial system [20]. The research team used an experimental design to explore these issues within a student population. A relationship was reported between the timing of a PTSD diagnosis being made and being offered treatment. Veterans whose diagnosis of PTSD was made after being arrested, but before being found guilty, were more likely to be referred for treatment than those whose diagnosis was made after being found guilty. This study could have important implications for those veterans involved in the judicial system who are also experiencing mental health difficulties.

Another paper published titled ‘The Link between PTSD and Functionality among United States Military Service Members Psychiatrically Hospitalized Following a Suicide Crisis’ reported the additive effect of psychiatric comorbidities alongside PTSD on functional impairment [21].

Also within this edition, Steven Koven published a meta-analysis of PTSD interventions [22]. This paper explores the existing knowledge around the effectiveness, or not, of current support for veterans with PTSD. A range of different treatments was reviewed, ranging from mindfulness to interventions that involved conjoint therapy working with the partners of veterans. One of the principal findings from this study was that the majority of treatment studies focus solely on reducing the burden of PTSD symptoms, with less attention given to supporting veterans to overcome the functional impairment associated with PTSD. For example, support back into employment or improving diminished social networks. This is a pertinent finding, as the data suggest that veterans with PTSD are at a high risk of social exclusion compared to those suffering from other mental health difficulties. One possible explanation for this could be that veterans typically take considerable time to seek help, which may lead to an erosion of their resources (e.g., social support) prior to engaging in treatment. As such, this points to the need to develop more holistic packages of support for veterans with PTSD.

It was our intention that readers of this special edition will be provided with new insights and will hopefully be inspired to contribute to the growing literature to help find better ways to support veterans with PTSD.
